# A misleading distal anterior cerebral artery aneurysm

**DOI:** 10.4103/2152-7806.69382

**Published:** 2010-09-16

**Authors:** Alexander G. Weil, Nancy McLaughlin, Paule Lessard-Bonaventure, Michel W. Bojanowski

**Affiliations:** Division of Neurosurgery, Department of Surgery, Notre Dame Hospital, University of Montreal Hospital Centre, Montreal, Quebec, Canada

**Keywords:** Pure acute subdural hematoma, aneurysm, anterior cerebral artery, callosomarginal artery

## Abstract

**Background:**

Aneurysmal rupture causing pure acute subdural hematoma (aSDH) is rare. In the four previously reported cases of distal anterior cerebral artery (ACA) aneurysm resulting in pure aSDH, blood distribution in the interhemispheric (IH) space has systematically incriminated the distal ACA as the source of rupture. We present a misleading case of a distal ACA rupture resulting in convexity aSDH with minimal IH blood.

**Case Description:**

A 51-year-old patient presented in coma with decerebrate posturing and a blown left pupil from a left convexity acute hemispheric subdural hematoma. She underwent urgent left craniectomy and subdural hematoma evacuation. Given the absence of identifiable etiology, including trauma, we performed an immediate postoperative Computed tomography-angiography (CTA) in order to rule out an underlying cause. The CTA revealed an aneurysm originating from the callosomarginal artery branch of the ACA. Although the minimal amount of IH blood and the remote distance of convexity blood from the aneurysm suggested that it may be a fortuitous finding, we considered the possibility that the two might be related. The patient underwent surgical aneurysm clipping, confirming that it had ruptured and allowing complete aneurysm obliteration. Following the procedure, the patient’s neurological and functional status gradually improved.

**Conclusion:**

Ruptured distal ACA aneurysms may present with convexity isolated aSDH with minimal IH blood. Quantity and distribution of isolated aSDH can be misleading and is not always a reliable predictor of aneurysm location. Misinterpretation of the aneurysm as an incidental finding would lead to improper management with potentially serious consequences.

## INTRODUCTION

Distal anterior cerebral artery (ACA) ruptured aneurysms presenting as isolated acute subdural hematoma (aSDH) are rare. In the literature, only four such cases have been reported, and in all the four there was a thick, wedge-shaped subdural blood clot in the interhemispheric (IH) fissure.[3,7,13] We report a case of a non-traumatic, isolated convexity aSDH from a ruptured distal ACA aneurysm with minimal IH blood. Knowledge of this occurrence may avoid misinterpretation of angiographic findings following spontaneous aneurysmal pure aSDH, thereby ensuring proper management of the aneurysm.

## CASE REPORT

**Figure 1:**
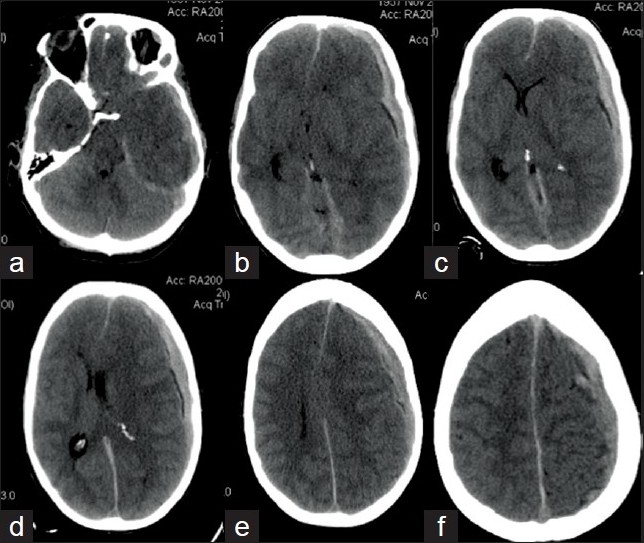
(a–f) Head CT showing left acute fronto-temporo-parietal subdural hematoma with mass effect, midline shift and transtentorial herniation

**Figure 2:**
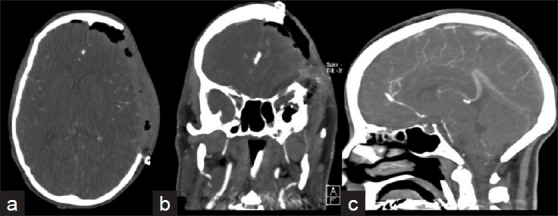
(a) Axial, (b) coronal, and (c) sagittal angio-CT performed immediately after surgery; an aneurysm pointing antero-superiorly and originating from the distal ACA (A3 segment) at its bifurcation with the callosomarginal artery was visualized

**Figure 3:**
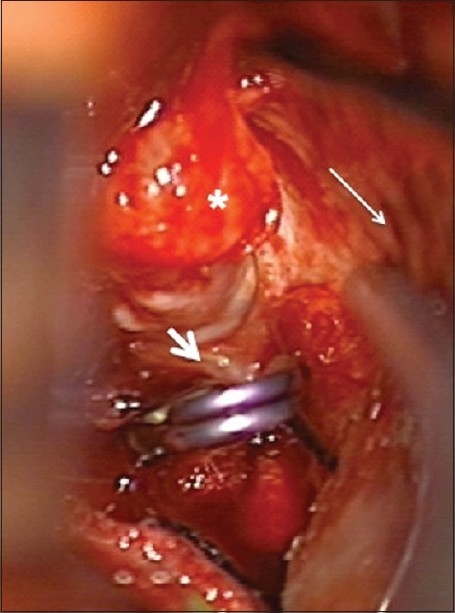
Intraoperative view through a left interhemispheric approach with the falx cerebri visualized on the right (long arrow). The site of rupture (asterix) is seen on the distal ACA aneurysm (short arrow). A temporary clip is placed on the ACA to allow dissection of the aneurysm.

**Figure 4:**
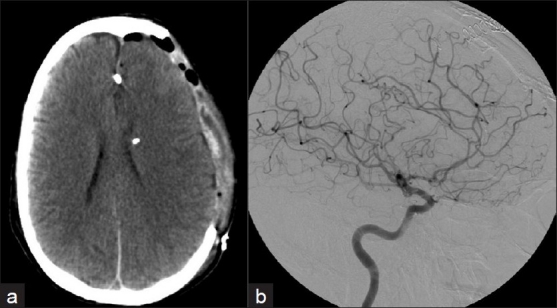
a) CT scan following aneurysm clipping through a frontal transcallosal approach; b) conventional cerebral angiogram through the left internal carotid artery shows complete aneurysm obliteration with preservation of the parent artery

A 51-year-old female patient with no previous past medical history presented with acute headache followed by rapid deterioration of her level of consciousness to coma with decerebrate posturing and a blown left pupil. There was no history of trauma or coagulopathy. Computed tomography (CT) showed a left convexity acute hemispheric subdural hematoma without intraparenchymal or intraventricular hemorrhage. There was doubtful subarachnoid hemorrhage [Figure 1]. She underwent an urgent left craniectomy, subdural hematoma drainage and insertion of an intracranial pressure (ICP) monitor. Given the absence of etiology, including trauma, we performed an immediate postoperative CT-angiography (CTA) in order to rule out an underlying cause. This study revealed an aneurysm of the callosomarginal artery branch of the ACA [Figure 2]. Although we could have interpreted this aneurysm as being fortuitous because of the minimal amount of IH blood combined with its remote distance from the convexity subdural hematoma, we considered the possibility that the two might be related. The patient underwent surgical aneurysm clipping through a frontal IH approach, confirming that it had ruptured [Figure 3] and allowing complete aneurysm obliteration [Figure 4]. Following the procedure, the patient’s neurological and functional status gradually improved.

## DISCUSSION

Pure aSDH without SAH caused by aneurysmal rupture is very rare and has only been described in 31 other cases in the literature.[1–5,7–13] The most frequently reported site of aneurysm location is the posterior communicating artery (50%, *n* = 15). Other locations include the middle cerebral artery (16.7%, *n* = 5), anterior communicating artery (13.3%, *n* = 4), internal carotid artery (3.33%, *n* = 1) and angular artery (3.3%, *n* = 1).[1,2,4–6,8–13] Four cases of distal ACA aneurysms have been reported [Table 1]. In all four of these, a 1-cm thick, wedge-shaped blood clot in the IH space was present in addition to convexity SDH.[3,7,13] Therefore, blood is expected to be found in the IH space when pure aSDH is caused by a distal ACA aneurysm rupture.

**Table 1:** Cases of pure aSDH caused by ruptured distal ACA aneurysms

Case	Author	Year	Demographic	Presentation	CT scan: aSDH	Distal ACA aneurysm site	Treatment	Outcome
1	Watanabe	1991	51, M	Comatose	Convexity and thick interhemispheric	Pericallosal-callosomarginal	Evacuation and clipping	Death
2	Hatayama	1994	55, M	Comatose	Convexity and thick interhemispheric	NA	Evacuation and clipping	Good
3	Hatayama	1994	66, F	Comatose	Convexity and thick interhemispheric	NA	Evacuation and clipping	Handicap
4	Katsuno	2003	63, F	Headache, N	Convexity and thick interhemispheric	Pericallosal-callosomarginal	Evacuation and clipping	Good

NA, not available; N, nausea; M, male; F, female

Our case of distal ACA rupture is misleading because the aneurysmal rupture led to convexity aSDH with minimal IH blood. There is always the possibility that a ruptured aneurysm could have led to fall, which in turn gave rise to a secondary convexity subdural bleed. However, this does not apply in our case since our patient did not experience a fall nor a head trauma. There is also the possibility that the aneurysm was an incidental finding during the investigation of a spontaneous aSDH. However, intraoperative findings clearly confirmed that the aneurysm bled in this patient in whom no other cause or risk factor could be identified. This case demonstrates that, as with subarachnoid hemorrhage, we cannot always rely on the location of the subdural hematoma to exclude the possibility that it is caused by a ruptured aneurysm.[6] Misinterpretation of the aneurysm as an incidental finding would have led to improper management with potentially serious consequences.

The mechanism underlying aneurysmal bleed into the subdural space is based on aneurysmal rupture across the arachnoid membrane, either from a high pressure bleed or from adhesion of the aneurysm to the arachnoid and/or dura from prior bleeds resulting in an extension of the aneurysm into the subdural space.[9,14] Most of the blood in reported cases of pure aSDH is found in the subdural space in close proximity to the aneurysm itself.[1–5,7–13] We hypothesize that, in our case, the rigidity of the falx cerebri, as well as the restricted area between the two hemispheres, forced the blood from the aneurysm toward the convexity, where it accumulated and in turn transmitted the pressure toward the midline.

The disability and mortality rates are high in pure aSDH from ruptured aneurysms.[3,9,10,13] In order to maximize positive outcome, it is important to consider all possibilities even in the presence of a misleading CT scan.

## CONCLUSION

Ruptured distal ACA aneurysms may present with convexity isolated aSDH with minimal IH blood. Quantity and distribution of isolated aSDH can be misleading and is not always a reliable predictor of aneurysm location.
